# Eosinophils as Drivers of Severe Eosinophilic Asthma: Endotypes or Plasticity?

**DOI:** 10.3390/ijms221810150

**Published:** 2021-09-21

**Authors:** Glenn Van Hulst, Fabrice Bureau, Christophe J. Desmet

**Affiliations:** 1 Laboratory of Cellular and Molecular Immunology, B34, GIGA Institute and Faculty of Veterinary Medicine, Liège University, 4000 Liège, Belgium; gvanhulst@uliege.be (G.V.H.); fabrice.bureau@uliege.be (F.B.); 2 Walloon Excellence in Life Sciences and Biotechnology (Welbio), 1300 Wavres, Belgium

**Keywords:** eosinophilic asthma, eosinophils, endotypes, plasticity, eosinophil subsets, immunotherapy

## Abstract

Asthma is now recognized as a heterogeneous disease, encompassing different phenotypes driven by distinct pathophysiological mechanisms called endotypes. Common phenotypes of asthma, referred to as eosinophilic asthma, are characterized by the presence of eosinophilia. Eosinophils are usually considered invariant, terminally differentiated effector cells and have become a primary therapeutic target in severe eosinophilic asthma (SEA) and other eosinophil-associated diseases (EADs). Biological treatments that target eosinophils reveal an unexpectedly complex role of eosinophils in asthma, including in SEA, suggesting that “not all eosinophils are equal”. In this review, we address our current understanding of the role of eosinophils in asthma with regard to asthma phenotypes and endotypes. We further address the possibility that different SEA phenotypes may involve differences in eosinophil biology. We discuss how these differences could arise through eosinophil “endotyping”, viz. adaptations of eosinophil function imprinted during their development, or through tissue-induced plasticity, viz. local adaptations of eosinophil function through interaction with their lung tissue niches. In doing so, we also discuss opportunities, technical challenges, and open questions that, if addressed, might provide considerable benefits in guiding the choice of the most efficient precision therapies of SEA and, by extension, other EADs.

## 1. Introduction

Asthma is a highly prevalent chronic respiratory disease responsible for a considerable health burden worldwide [1]. As generally defined by the Global Initiative on Asthma (GINA), asthma is “a heterogeneous disease, usually characterized by chronic airway inflammation. It is defined by the history of respiratory symptoms such as wheeze, shortness of breath, chest tightness and cough that vary over time and in their occurrence, frequency and intensity, together with variable expiratory airflow limitation” [2]. Inflammation of the asthmatic airways promotes mucus production, remodeling of the airway wall, and bronchial hyperresponsiveness (BHR), which originates from the increased reactivity of smooth muscle cells to a non-specific stimulus such as cold air. Asthma symptoms typically arise in episodes, with intermittent periods of more severe and sustained worsening of symptoms known as exacerbations [3]. Mild asthma can usually be controlled adequately using medications that target the inflammatory component of the disease, such as inhaled corticosteroids, or that restore airflow, such as short- or long-acting β2-adrenergic agonists [4]. Yet, standard of care treatments provide insufficient control over the disease in 5–10% of cases said to suffer from refractory, uncontrolled, or severe asthma, which accounts for about half of the asthma-related economic costs [5,6].

A very important component of the latest GINA definition is the recognition of asthma as a heterogeneous disease [2]. In spite of convergent respiratory symptoms, extensive stratification studies showed that asthma encompasses a variety of phenotypes that may be driven by different underlying pathophysiological mechanisms called endotypes [7,8]. Broad separation of asthma phenotypes can be made today based on a combination of parameters, including age of disease onset (childhood-onset asthma (COA) versus adult-onset asthma (AOA)), decline or preservation of lung function (based on forced expiratory volume in 1 second (FEV1)), and atopic (viz. the genetic predisposition to an allergy) or allergic status [7]. In direct support of the existence of endotypes, asthma is also often broadly dichotomized into type-2 high (T2-high) and non-type 2 (non-T2, or T2-low) phenotypes [9,10]. This latter dichotomy refers to the involvement of type-2 helper T (Th2) lymphocytes or at least their hallmark signature cytokines in driving T2 asthma symptoms. Th2 cells are professional producers of key cytokines involved in allergy and type-2 inflammation, including interleukin (IL)-4 and IL-13 which, among other actions, promote BHR and the production of IgE and mucus, as well as IL-5, which promotes eosinophilia [11,12]. Because of the relatively easy accessibility of blood and induced sputum, patient stratification between T2 and non-T2 phenotypes is often based on the presence or absence of blood or airway eosinophilia [13,14]. In non-T2 asthma, eosinophilia is absent, and there is no prominent sign of activation of the type-2 immune pathways [9,10]. A large majority of children and approximately half of the adults with asthma have typical allergy-driven T2 allergic asthma, characterized by blood and lung eosinophilia and elevated serum IgE or positive skin prick tests for common environmental allergens, such as house dust mite antigens [15]. Yet, asthma with prominent T2 features, notably eosinophilia, can also develop in the absence of signs of allergies, such as IgE reactivity to allergens. These patients, most often adults with AOA, are classified as having T2 high non-allergic (or intrinsic) eosinophilic asthma [16].

Eosinophils currently occupy the central stage in asthma management. The realization that asthma and especially its most difficult cases are highly heterogeneous in terms of phenotype and mechanisms of disease has drastically switched therapeutic approaches from a “one fits all” strategy toward precision medicine [7,8,17]. As such, adequate phenotyping and endotyping of individual asthma patients is becoming paramount in guiding optimal therapeutic choices [18]. In this line of thought, eosinophilia is used as a useful biomarker of T2 asthma [19]. Additionally, eosinophils have also become the target of choice in severe eosinophilic asthma (SEA), as demonstrated by the beneficial effects of eosinophil-targeting immunotherapies on disease exacerbations [20,21]. These “anti-eosinophil” biological therapies exploit the dependency of eosinophilia on IL-5. One class relies on monoclonal antibodies that neutralize circulating IL-5, called mepolizumab and reslizumab [22,23], which limit but do not eliminate eosinophil development in the bone marrow [24]. The other class uses a monoclonal antibody called benralizumab, which binds the alpha subunit of the IL-5 receptor (IL5Rα) on the cell surface and elicits antibody-dependent cellular cytotoxicity [25], often leading to a complete depletion of eosinophils and their progenitors [26].

Nonetheless, anti-eosinophil biological treatments mostly provide clear benefits specifically in SEA, but not in non-T2 asthma [27], indicating that eosinophils are not systematically important drivers of asthma symptoms. In addition, recent analyses suggest that anti-eosinophil therapeutics on average provide more marked benefits in terms of reduction in exacerbation rates and improvement of lung function in SEA patients with AOA compared with COA [28,29,30]. Clinical findings with anti-eosinophil therapeutics thereby suggest that eosinophils, even when infiltrating the lungs in significant numbers, may have a variable impact on asthma manifestations, depending on the disease phenotype or endotype. These notions are in line with the broader realization that predicting the role of eosinophils and their response to anti-eosinophil therapies in human “eosinophil-associated” diseases (EADs) characterized by tissue eosinophilia remains a challenge [20,21]. This calls for a deeper understanding of eosinophil biology in EADs, including SEA, by addressing fundamental questions pertaining to the determinants of the pathogenic roles of eosinophils.

In this review, we address our current understanding of the role of eosinophils in asthma. We further address the possibility that different SEA phenotypes may be driven by differences in eosinophil biology through eosinophil “endotyping” or plasticity. Finally, we discuss the technical challenges and open questions that, if addressed, could provide considerable benefits in guiding the choice of the most efficient precision therapies of SEA and, by extension, of other EADs.

## 2. Eosinophils as Evolving “Usual Suspects” in Asthma

Eosinophils are a specialized type of granulocytes most often characterized in different species by their distinctive red or pink specific granule staining under the action of acidic dyes in bright field microscopy [31]. Eosinophils are an ancient evolutionary innovation, dating back at least 350 million years, since this specialized cell type is present in all vertebrate lineages [32]. In spite of this conservation being highly suggestive of important functions, the true biological roles of eosinophils remain elusive [21,33,34]. The classical paradigm states that eosinophils have evolved under the selective pressure of parasitic worms, which they can kill in different settings. This view is, however, debated [35], not the least because of the observation that patients under anti-eosinophil therapy do not seem to be more at risk of worm infection in their endemic regions [36,37]. More recently, especially in the context of the COVID-19 pandemic, a putative role of eosinophils in antiviral protection is gaining some momentum [38]. In addition, eosinophil eradication with benralizumab seems to be associated with increased risk of bacterial infection-induced asthma exacerbations [39]. Eosinophils also associate with tuberculous granulomas in humans and help control tuberculosis infection in mice [40]. This illustrates that there may be mounting support to the hypothesis that eosinophils play innate immune protective roles that are not entirely redundant with those of other innate immune cells. Aside from immune functions, eosinophils have also been attributed roles in tissue morphogenesis, mucosal homeostasis and metabolism, although evidence so far mostly comes from preclinical models [33,34,41]. Perhaps most compellingly in our current state of knowledge, eosinophils appear to be important effectors of type-2 immune processes which, if they are traditionally associated with anti-parasitic and anti-toxin defense, are also essential in tissue repair and remodeling [35,42].

Notwithstanding the protective or beneficial roles eosinophils may have played in our long ancestry—and are likely still playing today—eosinophils are mostly studied in the context of EADs [21]. EADs have been rising steadily over the last few decades, with T2 or eosinophilic asthma being one of the most prevalent and best-studied examples. Steady state eosinophils are a rare group of immune cells that only represent 1–3% of all peripheral blood leukocytes, or around 100 cells per cubic millimeter of blood on average. In contrast, in patients with T2 asthma, eosinophil numbers increase above this threshold in a process known as eosinophilia [43]. A correlation was found between blood eosinophilia and asthma severity [44,45,46,47], prompting the development of eosinophil-targeting biological therapies. Eosinophilia results from the stimulation of eosinophil production from hematopoietic stem and progenitor cells through complex differentiation processes that are only partially resolved [48,49,50]. The promotion of eosinophilia involves signaling by IL-3, IL-5, and GM-CSF, of which the receptors share the CSFR2B cytokine receptor common subunit. Of these three cytokines, IL-5 definitely plays the most critical role. Indeed, genetic ablation of IL-5 or its neutralization with monoclonal antibodies abolishes eosinophilia in preclinical models and human patients [36,51]. IL-5 is nonetheless not entirely indispensable for eosinophil development, as Il5-deficient mice and human patients receiving anti-IL-5 therapy all retain residual eosinophils [34,50].

The study of eosinophils has long suffered from prejudice due to their apparent proximity with neutrophils. Like neutrophils, they are classically viewed as terminally differentiated and short-lived once they exit the bone marrow. Eosinophils are therefore mostly envisioned as circulating “sentinels” that invade tissues following immune detection of pathogenic threats. This view is, however, to be mitigated, as significant populations of eosinophils are present in homeostatic conditions in various human organs, including the lungs, and eosinophils may be involved in tissue morphogenesis [41]. In response to stimuli such as GM-CSF, eosinophils can survive for weeks, indicating that in contrast with neutrophils, they should not be considered short-lived cells [51].

Tissue recruitment of eosinophils involves the activation of their surface integrins in response to chemotactic cues including eotaxins and lipid mediators, inducing their extravasation in target tissues [41]. Once infiltrated, eosinophils may undergo activation, which is when they are expected to become a liability. Recent years have, in this regard, revealed a surprising variety of eosinophil activities in response to activation. These activities extend beyond the classical view of eosinophils “merely” releasing their specific toxic granule contents, comprising eosinophil cationic protein (ECP), eosinophil-derived neurotoxin (EDN), eosinophil peroxidase (EPX), and major basic protein (MBP), in order to kill invaders or accidentally cause undue tissue damage in EADs. First, eosinophils may undergo three different types of degranulation (reviewed in [52]). These include classical and compound exocytosis, which lead to the release of the full granule content, as well as piecemeal degranulation, a more controlled form of mediator release occurring through so-called sombrero vesicles or exosomes [53]. Aside from that, activated eosinophils can undergo cytolysis, a non-apoptotic form of cell lysis. Cytolysis may release cell-free granules that are surprisingly long-lived and still capable of stimulus-induced responses [54]. Cytolysis also releases the large amounts of galectin-10 contained in human eosinophils, which subsequently crystallizes into Charcot–Leyden crystals that can enhance type-2 pathology [55]. Finally, eosinophils can also release eosinophil extracellular traps composed of mitochondrial DNA and granule proteins (EETosis) [56]. In addition to their signature mediators, activated eosinophils are also an important source of cytokines and growth factors [57]. Among these factors are several key actors of type-2 immunity and tissue remodeling with a suspected role in asthma, most notably IL-4, IL-13, and transforming growth factor (TGF)-β1.

In line with their revised role as regulators of tissue morphogenesis and repair, several lines of evidence suggest that eosinophils actively contribute to the airway remodeling process in asthma. Airway remodeling refers to structural changes of the airway walls as a result of repeated injury and repair processes. In asthma, airway remodeling is characterized by increased airway smooth muscle cell mass, epithelial cell hyperplasia, goblet cell metaplasia, and thickening of the reticular basement membrane (RBM) by deposition of collagen, tenascin, and other matrix proteins, leading to a progressive loss of lung function [58]. Notably, thickening of the RBM is more reported in eosinophilic asthma compared with non-eosinophilic asthma [43]. The precise mechanisms underlying airway remodeling processes in eosinophilic asthma are not yet fully understood. However, early findings showing elevated levels of TGF-β1 in the asthmatic airway suggest a possible role for eosinophils [59]. Eosinophils are a major source of TGF-β1 and are able to release it at the site of allergic inflammation [60,61]. Bronchial biopsies from atopic asthma patients treated with anti-IL-5 (mepolizumab) showed an association between reduced airway eosinophilia and the significant reduction of biomarkers for airway remodeling such as RBM collagen, tenascin, and lumican deposition [62]. Reduction of these airway remodeling biomarkers also correlated with a proportional reduction of eosinophils and TGF-β1 concentrations in bronchoalveolar lavage. In addition, transgenic overexpression of IL-5 in murine lungs increased eosinophil infiltration in the airway lumen and induced pathological changes to the lung epithelial characteristic of airway remodeling in asthma [63]. Other cytokines linked to airway remodeling and expressed by eosinophils are heparin-binding epidermal growth factor (HB-EGF), nerve growth factor (NGF), TGF-α, and Th2 cytokines IL-4 and IL-13 [64].

Altogether, the “built-in” activities of eosinophils have contributed to making them strong suspects in asthma pathophysiology, prompting the development of specific “anti-eosinophil” biologicals.

## 3. Refining the Role of Eosinophils in Asthma through Eosinophil-Targeting Biological Therapies

While eosinophils have long been “usual suspects” in asthma, anti-eosinophil biologicals offer unique opportunities for directly establishing their level of involvement in disease manifestations. In this regard, clinical trials of anti-eosinophil biological therapies revealed that the role of eosinophils in asthma is more complex than anticipated [65]. Puzzlingly, the first efficacy trials of mepolizumab failed to detect the expected clinical benefits for BHR or lung function [27,66]. A first trial in allergen-challenged mild allergic asthmatics did not detect any effect of mepolizumab on BHR, in spite of an efficient reduction in blood eosinophils [66]. A second trial in well-controlled mild asthmatic patients also did not detect significant improvement in lung function (FEV1, peak expiratory flow rate), use of β2-agonists, or general disease control (summary symptom score, asthma quality of life questionnaire) over a 20-week treatment course, yet it still efficiently reduced blood and sputum eosinophilia [27]. In this same trial, exacerbation rates were not significantly different in the treated patients compared with a placebo group. Nonetheless, with the recruited patients being mild asthmatics, the analyses on exacerbations were underpowered, and a trend toward a decrease in the exacerbation rate was noted in patients receiving mepolizumab.

It therefore appeared from early trials that eosinophils were not systematic direct drivers of BHR and airflow limitation, raising the question of their actual contribution, if any, to asthma symptoms. At about the same time, other trials established a strong correlation between eosinophilia and poor asthma control and exacerbation rates [45,46,47]. These observations strongly suggested that anti-eosinophil therapeutics should be targeted toward patients with SEA, primarily to control exacerbations. At last, subsequent trials with mepolizumab, reslizumab, and benralizumab confirmed that all three biologicals, if targeted to SEA patients, efficiently reduced SEA exacerbations [22,23,67]. Anti-eosinophil therapeutics thereby firmly established eosinophils as key actors in SEA exacerbations. In addition, several studies reported improvement in lung function (FEV1) following anti-eosinophil therapy in SEA [30,68,69].

In spite of the progress allowed in SEA management, not all patients respond equally well to anti-eosinophil therapeutics. Further studies therefore aimed at determining which factors may influence the response to anti-eosinophil treatments [18]. Perhaps not surprisingly, given their targeting toward SEA, a history of frequent previous exacerbations and blood eosinophilia were consistently identified as good predictors of the response to all three anti-eosinophil therapeutics [22,23,30,70,71,72,73,74,75]. T2-high asthma therefore appears to correspond to the endotype most responsive to anti-eosinophil biologicals. Further post-hoc stratification of the response of SEA patients according to other disease phenotypic traits failed to detect an effect of the atopic status, IgE serum concentrations, allergy to fungal antigens or house dust mites [74,76,77,78,79], or of body mass index [74,80]. A generally better response was, however, noted with benralizumab and reslizumab for patients with AOA compared with those with COA in terms of the reduction of exacerbations and improvement of FEV1 [28,29,30]. Interestingly, this better response of adult-onset SEA patients still remained, or was even more prominent, when patients in the COA and AOA groups were first selected for blood eosinophil counts >300 cells/µL [29,30]. A similar trend was observed in a post hoc analysis of the SIROCCO and CALIMA randomized control trials when focusing on patients with blood eosinophil counts >300 cells/µL and stratified based on the presence or absence of fixed airway obstruction (FAO) [69]. Patients with FAO benefited from a greater reduction in severe exacerbations, higher increase in FEV1, and better patient-reported outcomes and symptom improvements following benralizumab therapy. Finally, still based on the initial selection of patients with blood eosinophil counts >300 cells/µL, a greater benefit of mepolizumab in the MENSA trial was described in patients with non-atopic asthma compared with those with atopic or strongly atopic asthma [79].

Altogether, current clinical data suggest a generally stronger response to anti-eosinophil therapy in T2-high, possibly non-atopic, adult-onset SEA with degraded lung function. Part of the explanation to the above is probably that patients with adult-onset SEA or with FAO start “worse off” or have more room for improvement, as they tend to experience more severe exacerbations and more degraded lung function before the initiation of anti-eosinophil therapy [69]. Nonetheless, this still raises the question of what makes eosinophils especially detrimental in particular asthma phenotypes.

It is to be expected that a significant part of the benefit of anti-eosinophil therapeutics simply arises from the decrease in (or full depletion of) circulating eosinophils, dampening their subsequent accumulation in the airways. Nonetheless, eosinophilia alone does not seem sufficient to fully explain how eosinophils cause more frequent, more severe exacerbations and negatively affect lung function in specific SEA phenotypes. An intriguing observation of early randomized clinical trials with mepolizumab is that similar reductions in exacerbations are obtained with a range of mepolizumab doses, even though a significant population of residual sputum eosinophils persists in patients receiving lower doses of therapy [22]. In addition, eosinophilic exacerbations (sputum eosinophils >2%) still make up to 50% of residual asthma exacerbations in patients receiving mepolizumab, in spite of marked reductions in blood eosinophil counts [81]. It was also noted that mepolizumab does not alter the expression of activation markers on residual lung tissue eosinophils [82], nor does it elicit any detectable transcriptional alterations in blood eosinophils [83]. In addition, the risk for SEA exacerbations is better predicted with a combination of elevated blood eosinophil counts and elevated fractional exhaled nitrogen oxide (FeNO) than either parameter alone [73,84], and elevated FeNO levels are good predictors of eosinophilic exacerbations in mepolizumab-treated patients [81]. FeNO is mostly produced in response to IL-13-stimulated production of inducible nitric oxide synthase in lung epithelial cells and is often considered a good surrogate of ongoing type-2 inflammation in asthma [85]. Taken together, current evidence suggests that in some SEA patients, especially those most likely to strongly benefit from anti-eosinophil therapeutics, particular type-2 processes may make eosinophils more prone to being recruited to their airways or to promoting exacerbations and lung function decline.

**Figure 1:**
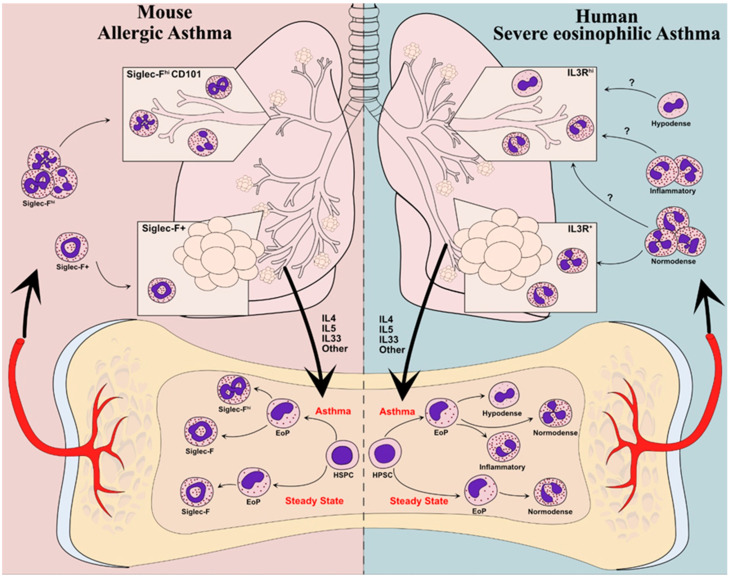
Developmental “endotyping” of murine and human eosinophils in asthma. In mice (**left** panel), eosinophils in the steady state populate the lung parenchyma but do not infiltrate the bronchial mucosa. In models of allergic asthma, type-2 inflammatory cues, including IL-5, promote the differentiation of eosinophil progenitors (EoPs) into large numbers of “inflammatory” SiglecF^hi^ eosinophils that infiltrate the bronchial mucosa. In humans (**right**), patients with severe eosinophilic asthma harbor hypodense eosinophils that are most likely derived from the altered development of eosinophil progenitors in response to inflammatory cues. Whether other eosinophils with an “inflammatory” program develop as in mice remains to be established. The respective contribution of normodense, hypodense, and putative inflammatory eosinophils to mucosal inflammation in human asthma remains to be established. (HSPC: hematopoietic stem and progenitor cell; EoP: eosinophil progenitor).

This raises a series of interconnected questions. First, what are the particular processes in SEA patients driving eosinophil recruitment and activation of their detrimental activities? Second, what are the eosinophil activities most relevant to their pathogenicity in SEA? Third, are these differences in eosinophil pathogenicity induced at the level of the lung tissue through local “plasticity” of eosinophils, or do they rather (or also) arise systemically within eosinophils themselves through what we refer to below as eosinophil “endotyping” (Figure 1)? Answering these questions will require more detailed comparisons of eosinophil activities in different asthma phenotypes in patients and preclinical models, and it will likely require the power of novel technologies. Because these questions are interconnected and have eosinophils at their core, we will address possible avenues for future research through the third “eosinophil-centric” question pertaining to eosinophil endotyping or plasticity.

## 4. Eosinophil Endotypes or Local Plasticity as Potential Effectors in SEA

### 4.1. Emerging Evidence for Eosinophil “Endotypes”

Here, we introduce the concept of eosinophil “endotypes”, a term we derive from the adaptation of the asthma endotype concept. Like how endotypes of asthma refer to built-in differences in pathophysiological mechanisms of disease, eosinophil endotypes would refer to specific functional programs imparted to eosinophils (or subsets thereof) as part of their developmental process in specific contexts. In this model, (subsets of) eosinophils with specific activities different from those of “classical” (viz. steady state) eosinophils would arise as a consequence of pathology-associated signals perceived by the developing progenitors of eosinophils (Figure 1). Different eosinophil endotypes could differ, for instance, with regard to their recruitment capacity to target organs or their ability to exert pro- or anti-inflammatory activities following activation, as well as the level of such activities. One analogous known occurrence of such an “endotyping” process is, for instance, myeloid-derived suppressor cells (MDSCs). MDSCs develop from myeloid hematopoietic progenitors in chronic inflammation and cancer in response to cytokines such as GM-CSF, IL-6, and IL-10 [86,87]. When invading tissues, MDSCs exert immunosuppressive activities that widely differ from their “normal” monocyte and neutrophil counterparts, illustrating the profound effects that developmental endotyping can have on myeloid cells. Importantly, because they would be detectable in the patients’ blood, eosinophil endotypes, if they exist, may provide amenable biomarkers of disease phenotypes or predictors of the response to anti-eosinophil therapies and other interventions in SEA and other EADs. Actually, signs of eosinophil endotyping have already been reported in both mice and humans, as we elaborate below.

Eosinophil endotyping would occur during eosinophil development from their hematopoietic progenitors (Figure 1). Classically, eosinophils are considered to arise from IL5Rα-expressing eosinophil progenitors (EoPs) that belong to the common myeloid progenitor (CMP) or the (pre-)granulocyte macrophage progenitor (GMP) compartments in humans and mice, respectively [88,89]. Single-cell studies more recently suggested that eosinophils develop from Gata1-expressing hematopoietic stem and progenitor cells [48,90,91], which would counterintuitively make their usual developmental trajectory and that of basophils closer to that of megakaryocytes and erythrocytes than to that of monocytes and neutrophils [48,92,93]. Further complicating this picture, a recent report showed that IL5Rα^+^ Ly6G^+^ murine myeloid progenitors that normally give rise to neutrophils may express eosinophil genes in response to IL-5 ex vivo [94]. While the actual contribution of the latter specific differentiation program to eosinophil development in vivo remains to be established, this report suggests that eosinophil differentiation programs may be more flexible and possibly more “adaptable” than expected.

Clues of eosinophil endotyping in asthma have been reported in both humans and mice. In mice, only one subset of eosinophils is commonly detected in the blood and lungs in homeostasis. However, following the development of airway allergy to house dust mite antigens, two subsets of eosinophils become detectable in the blood and lungs of mice [95]. One of these subsets retains the characteristics of steady state eosinophils, including immunoregulatory activities [95,96], whereas the other acquires a more inflammatory gene expression profile. Both subsets differ by the expression of markers Siglec-F and CD101 [95,96]. This dichotomy between Siglec-F^+^ CD101^lo^ “regulatory” and Siglec-F^hi^ CD101^+^ “inflammatory” eosinophils is not specific to asthma. In transgenic mice overexpressing IL-5, Siglec-F^hi^ and Siglec-F^+^ eosinophil subsets with distinct gene expression profiles were detected in the bone marrow and blood [95]. “Regulatory” and “inflammatory” eosinophil subsets were also detected in the murine models of eosinophilic esophagitis [95], suggesting that inflammatory eosinophils could be a common feature of murine models of EAD. The process of eosinophil endotyping giving rise to either homeostatic or inflammatory eosinophils is very important in mice, as inflammatory eosinophils largely differ from their homeostatic counterparts in terms of pathophysiological activity [95,97,98].

The above observations in mice raise the question of the mechanisms leading to endotyping of inflammatory eosinophils during eosinophilopoiesis. IL-5 is expected to be an essential player in the process. Indeed, neutralization of IL-5 potently antagonizes inflammatory eosinophil development in mice [95]. In addition, the bone marrow of IL-5-transgenic mice readily develops inflammatory eosinophils, and its eosinophil progenitors harbor specific gene expression programs [99]. There are also indications that additional signals such as IL-4 may influence murine eosinophil development downstream of IL-5 [100]. It is therefore a likely possibility that inflammatory signals originating from the tissue or environment, including but probably not solely IL-5, may modulate eosinophil development in EADs to impart functions on (subsets of) eosinophils that differ from those of their steady state counterparts.

Evidence of eosinophil endotyping in humans is scarcer yet much more ancient. Studies in the 1980s reported the presence of hypodense eosinophils in asthmatic patients. Hypodense eosinophils segregate with mononuclear cells in density gradients, away from “classical” normodense eosinophils. Increased numbers of hypodense eosinophils are present in the blood, bronchoalveolar lavage, and lung tissue of SEA patients [99]. Even though these hypodense eosinophils were originally interpreted as activated eosinophils [101,102], it seems more likely that they arise from a specific differentiation program. Indeed, circulating hypodense eosinophils are characterized by smaller and less numerous granules [101] and display increased survival, adhesion, oxygen metabolism, superoxide production, and antibody-dependent cytotoxicity following activation compared with normodense eosinophils [102]. This would not be expected if hypodense eosinophils already underwent activation. These latter features actually resemble those of human hypodense neutrophils, which play important roles in human disease, and suggest a similar type of developmental programming [103,104]. Reassessing human hypodense eosinophils and their responses to current biological treatments in SEA and other EADs using modern approaches could therefore be very timely and worthwhile.

As a cautionary note, we need to stress that the reciprocal translation of murine and human observations of eosinophil endotyping may not be entirely straightforward in our current state of knowledge [105]. We acknowledge that it may be tempting to directly relate murine inflammatory eosinophils to human hypodense eosinophils. Likewise, it may be tempting to expect that patients with EADs will harbor circulating “inflammatory eosinophils” that should be depleted by anti-IL-5 biologicals. We argue that these views may be oversimplified given the current gaps in our knowledge for both murine and human eosinophil biology. To illustrate this notion and point toward important factors to consider in future studies of eosinophil endotypes, we will resort to a recent study from our laboratory [83].

In this work, we compared the gene expression profiles of circulating blood eosinophils in healthy patients and allergic SEA patients receiving either mepolizumab (anti-IL-5) or omalizumab (anti-IgE) therapy. Mepolizumab treatment in SEA patients consistently leaves a residual population of eosinophils, representing approximately half the normal eosinophil blood counts in the general population [50]. We aimed to determine whether these residual eosinophils, deprived of IL-5 during their development, would harbor altered gene expression programs. We observed that there was virtually no difference in the gene expression program of residual eosinophils from mepolizumab-treated patients compared to the eosinophils of the healthy controls and of omalizumab-treated SEA patients in the steady state. The eosinophils from mepolizumab-treated patients also showed very minimal alterations in their response to ex vivo stimulation. Our study may therefore support the idea that eosinophil endotyping does not occur in SEA and that anti-IL-5 biologicals do not act by modulating this process. We would, however, advise caution and point toward “blind spots” that need to be considered by the field in general. Importantly, our study focused on residual eosinophils deprived of IL-5 during their development. It thus cannot be used to infer the effects that elevated levels of IL-5, along with other inflammatory signals, may have on eosinophil development in SEA or other EADs. In support of this view, we reported in the same study that the residual eosinophils in steady state IL-5-deficient mice were highly similar to their wild type counterparts, contrasting with the significant impact that elevated IL-5 levels have on murine eosinophil development and endotyping [96,98]. In this same line of thought, human blood eosinophils have been suggested to express specific gene expression programs in patients with overt hypereosinophilia, including in asthma [106]. Finally, our matched omalizumab control group contained patients with normal eosinophil blood counts or only mild eosinophilia at the time of the study. In this regard, an important potential blind spot in the study of human eosinophil endotyping is timing. Much like how MDSCs or hypodense neutrophils are reactive cell subtypes, the development and persistence of eosinophil endotypes must be controlled in a timely manner. In our study, the SEA patients we recruited were all well-controlled at the time of the study, including in our omalizumab-treated control group. It is, however, likely that eosinophil endotyping may become most prominent in humans at specific times, much like eosinophil endotypes are studied in well-timed murine models. Obviously, such preferential episodes in SEA would include exacerbations.

We must also consider a second common blind spot: only normodense eosinophils are usually recovered as part of human eosinophil purification procedures. Because the patients in our study had only mild eosinophilia and were well-controlled, we do not expect them to have harbored prominent populations of hypodense eosinophils. Yet, we argue that hypodense eosinophils may be important in our understanding of the pathogenic roles of eosinophils in SEA and EADs.

Third, our study only included allergic SEA patients for matching purposes with omalizumab-treated control patients. Yet, we speculated that asthma endotypes could also influence eosinophil endotyping. We expected that different SEA phenotypes could result in different signals being sent from the diseased lung to developing eosinophil progenitors, influencing their downstream pathogenic potential in tissues. As noted above, eosinophils appear to be particularly detrimental in adult-onset, possibly non-allergic, highly remodeling SEA. We therefore advocate that future studies should assess and compare the heterogeneity of blood (and possibly bone marrow) eosinophils of asthma patients with different phenotypes of SEA.

Finally, and most importantly, molecular analyses on eosinophils are still essentially performed on bulk eosinophils. Eosinophils are notably difficult cells to work with using current gold-standard molecular single-cell profiling techniques such as single-cell RNA sequencing. Yet, endotypes that would develop as subsets of total eosinophils (such as hypodense eosinophils) could easily go unnoticed using bulk analyses. Advanced molecular and phenotyping techniques such as scATAC-seq (a transposase-based accessibility assay) or single-cell DNASE1 hypersensitivity assays could provide alternative solutions if they prove applicable to eosinophils [95,107].

### 4.2. Local Eosinophil Plasticity

**Figure 2:**
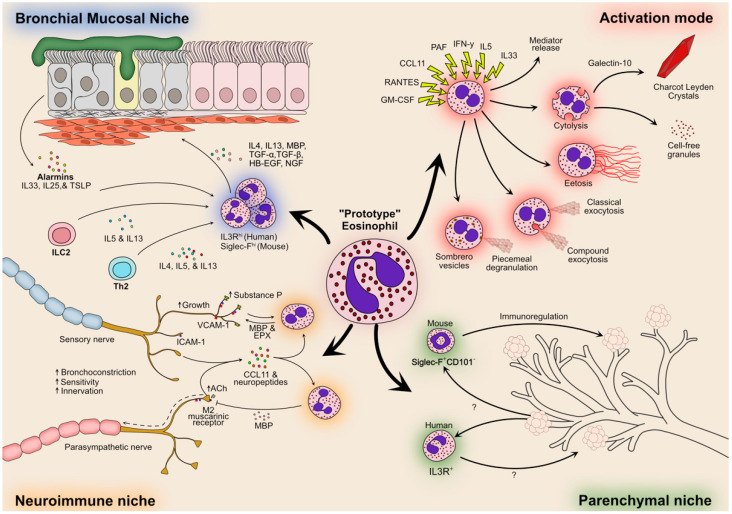
Lung niche-induced plasticity of eosinophils in asthma. Circulating eosinophils may be envisioned as “prototypes” that, once they reach different locations in the lung, occupy specific “niches” that will tailor their final effector functions. Three such potential niches are depicted here. The bronchial mucosa (upper left) is a preferential site of accumulation of eosinophils in asthma, in which eosinophils are exposed to many different clues they can perceive, such as type-2 cytokines and alarmins that may activate and modulate their function. In turn, eosinophils are induced to produce and release many mediators favoring airway remodeling. Eosinophils may also populate a more specific “neuroimmune” niche in the inflamed lung mucosa (lower left), as they are strongly attracted to autonomous nerves with which they tightly interact and whose function they modulate. In the steady state, eosinophils mostly populate the parenchymal lung compartment (lower right) and exert immunoregulatory functions (at least in mice). In these different niches, plasticity could also be involved in determining the type of activation and mediator release of eosinophils, influencing their impact on the surrounding tissue (upper right).

Eosinophil “endotypes” remain little-studied but need to be considered a possible explanatory factor with regard to subsequent eosinophil phenotypes in tissues. For instance, it has been proposed that lung Siglec-F^hi^ eosinophils derive from Siglec-F^+^ eosinophils in airway allergies through plasticity [107]. However, Siglec-F^hi^ eosinophils seem to readily develop in the bone marrow and circulate in allergic mice and in other models of eosinophilia [95,107]. The question therefore remains open as to whether homeostatic Siglec-F^+^ eosinophils may acquire a Siglec-F^hi^ phenotype through local plasticity in the lungs or if endotyping is solely responsible for the existence of the two murine eosinophil subsets. With this cautionary note given, here, we will consider a complementary source of human eosinophil diversity: local plasticity (Figure 2). Taking a known analogy again, the concept of eosinophil plasticity would resemble that of lung monocyte-derived macrophages. Classical monocytes can infiltrate the lung in the steady state or following immune stimulation. Once exposed to the specific lung context, these monocytic “prototypes” can differentiate into very distinct macrophage subsets such as interstitial or alveolar macrophages, which can themselves acquire distinct functional programs [108]. Translated to eosinophils, one may envision that local stimuli could modulate the activation program of recruited “prototypic” eosinophils and result in them acquiring distinct phenotypes and functions with direct and specific involvement in the ongoing physiological or pathological process [42]. The plasticity of lung immune cells may be influenced by virtually any cell type of their “niche” of residence, such as epithelial cells, fibroblasts, lymphocytes, neutrophils, or mast cells, all of which engage in crosstalk shaping the tissue responses.

The long-held view of eosinophils as invariant cells is already being extensively revised with the realization, initiated in mouse models, that eosinophils can be more diverse than initially anticipated [95,107,109]. There is also good evidence that human lung eosinophils may significantly differ in phenotype and behavior in different lung compartments and compared with their circulating homologues [107]. Older studies suggest tissue-resident eosinophils are more hypodense, express lower levels of the CD11b surface marker, and are less responsive to stimulation than blood eosinophils [109,110]. In addition, lung eosinophils were shown to locate mostly in the parenchyma in resected tumor margins from non-allergic non-asthmatic patients, whereas eosinophils populated both the parenchyma and bronchial walls in allergic asthmatic patients, reminiscent of the observations in mice [95]. In addition, tissue eosinophils from non-allergic non-asthmatic patients express lower levels of IL-3R and higher levels of CD62L compared with the sputum from allergic asthmatic patients [95].

Eosinophils are relatively easy to identify in mouse and human tissue using histological staining or flow cytometry, but assessing their relative adaptations to different tissue compartments or niches remains a challenge. As we discussed above, density and candidate cell surface marker approaches have revealed different eosinophil phenotypes. Yet, it is likely these different phenotypes themselves encompass eosinophils with different local adaptations. For instance, eosinophils have strong tropism toward lung nerve endings and can modulate nerve function and influence lung innervation during development and in asthma [111,112,113]. It is likely that lung eosinophils engage in crosstalk with neurons in what may be seen as a neuroimmune niche and undergo plastic adaptations in this process.

We expect that relating eosinophil plasticity to its pathological roles in SEA and EADs will require paying attention to some of the same “blind spots” discussed for eosinophil endotypes above, mainly timing and asthma phenotypes. Given the apparently more detrimental role of eosinophils in highly remodeling adult-onset SEA, understanding how eosinophils respond to the particular bronchial microenvironment during exacerbations in this asthma phenotype should also be highly prioritized. Toward this goal, and to generally decipher the role of eosinophils in EADs, we expect that understanding local plastic adaptations of eosinophils will require the power of novel techniques such as spatial transcriptomics or spatial epigenomics. In addition, special attention should be dedicated to different modes of eosinophil activation and cytolysis, which could also be influenced by endotyping and plasticity and be critical determinants of eosinophil function in SEA.

## 5. Conclusions

Eosinophils should no longer be considered short-lived, invariant, terminally programmed effector cells in asthma or EADs. As suggested by preclinical models, through observations in severe asthma and by the use of anti-eosinophil biologicals in SEA treatment, eosinophils are certainly more labile and adaptable than is envisioned in classical paradigms. Irrespective of the origin of the differences in the involvement of eosinophils in disease manifestations, be it endotyping or plasticity, we advocate that in-depth scrutiny of systemic and local eosinophil responses will provide insights critical for the understanding and management of eosinophilic asthma phenotypes and, by extension, of other EADs.

## Data Availability

Not applicable.
